# Deletion of Smooth Muscle Lethal Giant Larvae 1 Promotes Neointimal Hyperplasia in Mice

**DOI:** 10.3389/fphar.2022.834296

**Published:** 2022-01-24

**Authors:** Ya Zhang, Peidong Yuan, Xiaoping Ma, Qiming Deng, Jiangang Gao, Jianmin Yang, Tianran Zhang, Cheng Zhang, Wencheng Zhang

**Affiliations:** ^1^ The Key Laboratory of Cardiovascular Remodeling and Function Research, The State and Shandong Province Joint Key Laboratory of Translational Cardiovascular Medicine, Department of Cardiology, Chinese Ministry of Education, Chinese National Health Commission and Chinese Academy of Medical Sciences, Qilu Hospital, Cheeloo College of Medicine, Shandong University, Jinan, China; ^2^ Cardiovascular Disease Research Center of Shandong First Medical University, Central Hospital Affiliated to Shandong First Medical University, Jinan, China; ^3^ Department of Obstetrics and Gynecology, Liaocheng People’s Hospital, Liaocheng, China; ^4^ School of Life Science and Key Laboratory of the Ministry of Education for Experimental Teratology, Shandong University, Jinan, China; ^5^ Department of Cardiology, The First Affiliated Hospital of Zhengzhou University, Zhengzhou, China

**Keywords:** LGL1, STAT3, neointimal hyperplasia, smooth muscle, proteasomal degradation

## Abstract

Vascular smooth muscle cell (VSMC) proliferation and migration contribute to neointimal hyperplasia after injury, which causes vascular remodeling related to arteriosclerosis, hypertension, and restenosis. Lethal giant larvae 1 (LGL1) is a highly conserved protein and plays an important role in cell polarity and tumor suppression. However, whether LGL1 affects neointimal hyperplasia is still unknown. In this study, we used smooth muscle-specific LGL1 knockout (LGL1^SMKO^) mice generated by cross-breeding LGL1^flox/flox^ mice with α-SMA-Cre mice. LGL1 expression was significantly decreased during both carotid artery ligation *in vivo* and PDGF-BB stimulation *in vitro*. LGL1 overexpression inhibited the proliferation and migration of VSMCs. Mechanistically, LGL1 could bind with signal transducer and activator of transcription 3 (STAT3) and promote its degradation *via* the proteasomal pathway. In the carotid artery ligation animal model, smooth muscle-specific deletion of LGL1 accelerated neointimal hyperplasia, which was attenuated by the STAT3 inhibitor SH-4-54. In conclusion, LGL1 may inhibit neointimal hyperplasia by repressing VSMC proliferation and migration *via* promoting STAT3 proteasomal degradation.

## Introduction

Neointimal hyperplasia is a significant type of vascular remolding defined as the pathological accumulation of vascular smooth muscle cells (VSMCs) and extracellular matrix (ECM) in the intima. It is a process of excessive repair in the vascular wall caused by various activated cells and recycling substances responding to vessel injury (Zaman and Herath, 2008). Vascular injury inevitably occurs with various clinical procedures. Percutaneous coronary interventions such as balloon angioplasty and stents to treat ischemic coronary artery disease, vein grafts for coronary artery bypass graft surgery, and vascular access in hemodialysis can result in lumen re-narrowing and restenosis in a year after the operation (Schwartz et al., 1995; Roy-Chaudhury et al., 2001; Roy-Chaudhury et al., 2007; Harskamp et al., 2013; Byrne et al., 2017).

The behavior of VSMCs plays a vital role in neointimal hyperplasia. Gathering around the vessel lesion, activated inflammatory cells, and disturbed endothelial cells release a number of factors such as platelet-derived growth factor (PDGF) to stimulate the proliferation of VSMCs and subsequent migration from the media layer of the vessel to the intima (Dzau et al., 1991; Majesky et al., 1991; Nabel et al., 1993; Grant et al., 1994; Dzau et al., 2002). Previous studies have revealed that neointimal hyperplasia is regulated by many proteins including signal transducer and activator of transcription 3 (STAT3) (Dutzmann et al., 2015). When VSMCs are activated by cytokines or growth factors, STAT3 is phosphorylated and translocates into the nucleus to regulate the expression of target genes involved in proliferation and migration (Park et al., 2000). Earlier researchers found that blocking STAT3 by adenovirus-expressing domain-negative STAT3 or siRNA could inhibit VSMC proliferation and migration, thus decreasing neointimal formation in models of carotid balloon injury or jugular vein-carotid artery bypass (Shibata et al., 2003; Wang et al., 2007; Sun et al., 2012). Similarly, the administration of STAT3 inhibitor downregulated its activity and suppressed VSMC proliferation and migration in neointimal hyperplasia (Lim et al., 2007; Daniel et al., 2012). Although the role of STAT3 in neointimal hyperplasia is clear, the regulation of STAT3 expression and activity needs further exploration.

Lethal giant larvae (LGL) proteins are a group of highly conserved proteins first discovered in Drosophila (Grifoni et al., 2004). LGL1 and LGL2 are two homologs in mammals (Russ et al., 2012). LGL1 maintains cell polarity and acts as a tumor suppressor (Bilder et al., 2000; Kuphal et al., 2006; Tian and Deng, 2008; Lu et al., 2009). In our recent study, LGL1 could inhibit vascular calcification *via* high mobility group box 1 (Zhang et al., 2020). Nonetheless, the role of LGL1 in neointimal hyperplasia after the vascular injury has not been elucidated.

Here, we used smooth muscle-specific LGL1 knockout (LGL1^SMKO^) mice to explore the function of smooth-muscle LGL1 in neointimal hyperplasia. We found that LGL1 could inhibit neointimal hyperplasia after injury *via* STAT3.

## Materials and Methods

### Reagents

Adenovirus-expressing LGL1 and its control green fluorescent protein (GFP) were purchased from Vigenebio (Maryland, United States). Recombinant human PDGF-BB was from Proteintech (Wuhan, China). 3-Methyladenine (3-MA), MG132 and SH-4-54 were from Selleck Chemicals (Shanghai, China). SH-4-54 could effectively inhibit the phosphorylation of STAT3. The IC50 of SH-4-54 to STAT3 is 300 nM (Kd). Chloroquine (CQ) was from MCE (Shanghai, China).

### Mice

Smooth muscle-specific LGL1-knockout (LGL1^SMKO^) mice were generated by cross-breeding LGL1^flox/flox^ mice (Klezovitch et al., 2004) with transgenic Cre mice controlled by α-smooth muscle actin (α-SMA) promoter (Wu et al., 2007) as described (Zhang et al., 2020). Littermate LGL1^flox/flox^/Cre- mice were used as controls (CTR). All mice were bred at a constant temperature of 25°C and under a 12-hr-day/night light cycle. All animal experiments were performed with the protocols approved by the Animal Care and Use Committee of Shandong University.

### Animal Model for Neointimal Hyperplasia

We used common carotid artery (CCA) ligation to induce neointimal hyperplasia as reported previously (Wu et al., 2019). Briefly, 8-week-old mice were anesthetized with an intraperitoneal injection of sodium pentobarbital (40 mg/kg). A 10–15-mm median incision was made, and bilateral CCAs were carefully separated from veins and nerves. After exposing the left CCA, the ligation was performed below the bifurcation with 6–0 silk suture, above which are internal and external carotid arteries. The right CCA as a sham was processed as for the left CCA except for ligation. The neck incision was sutured, and animals were resuscitated in a warm and clean condition. After 3 weeks, mice were euthanized to collect tissues.

### Primary Culture of VSMCs

Mice at 4–6 weeks old were euthanized with sodium pentobarbital (40 mg/kg). Aortas were isolated and placed in culture dishes containing phosphate buffered saline (PBS). After dissection of extravascular connective tissues and adventitia, VSMC-enriched tunica media was transferred into tubes with cell culture medium and then cut into about 1 × 1-mm^3^ pieces. The tissue suspension was smeared evenly on the bottom of the culture bottle, which was inverted in a humidified incubator at 37°C and 5% CO_2_ for 2 h until small blocks adhered firmly to the surface. Adequate cell culture medium containing 15% fetal bovine serum, 100 µg/ml streptomycin and 100 U/mL penicillin was supplied, and bottles were turned over in the incubator to observe crawling cells 5–7 days later. When cell confluency reached about 80–90%, cells were passaged and plated for further use.

### Western Blot Analysis

The RIPA buffer (Solarbio. R0010) was used to extract proteins from cells and tissues. Proteins were fractionated by SDS-PAGE gel and transferred to PVDF membranes. After blocking with 5% skim dried milk/TBST for 1 h, membranes were incubated overnight at 4°C with primary antibodies, washed with TBST, incubated with corresponding secondary antibodies and observed by enhanced chemiluminescence (Pierce). The primary antibodies used were LGL1 monoclonal antibody (mAb) (CST, 12159s), GAPDH mAb (CST, 5174s), β-tubulin mAb (CST, 5568s), Cyclin D1 (CST, 55506s), proliferating cell nuclear antigen (PCNA) mAb (CST, 13110s), STAT3 mAb (CST, 9139s), and P-STAT3 (Y705) mAb (CST, 9145s). ImageJ software was used for analysis. All experiments were performed at least three times.

### Co-Immunoprecipitation

VSMCs were lysed with lysis buffer (Beyotime, P0013) and incubated with IgG or anti-LGL1 antibody at 4°C overnight to form an antigen-antibody complex. Then protein A/G magnetic beads were added (MCE, HY-K0202). After washing and magnetic separation, the precipitation was dissolved with 1 × SDS loading buffer for western blot analysis.

### Quantitative Real-Time Polymerase Chain Reaction

Total RNA was extracted from VSMCs and tissues by using an RNAfast200 kit (Fastagen, 220011), then a PrimeScript RT reagent kit (Takara, RR0037A) was applied to reverse transcript total RNA to complementary DNA. PCR amplification was performed with the SYBR Premix Ex Taq (Takara, RR420A). The primers’ sequences were as follows. LGL1: 5′-TAC​TGT​GAT​CAG​CCC​AAG​ACT​G-3′ and 5′- GGA​GGA​TCC​CAA​GAT​AGA​GGA​C-3′. GAPDH: 5′- GCA​CCG​TCA​AGG​CTG​AGA​AC-3′ and 5′-TGG​TGA​AGA​CGC​CAG​TGG​A-3′. Cyclin D1: 5′-AGG​CGG​ATG​AGA​ACA​AGC​AG-3′ and 5′- AGA​AAG​TGC​GTT​GTG​CGG​TA-3′. PCNA: 5′-TAC​AGC​TTA​CTC​TGC​GCT​CC-3′ and 5′-TTT​TGG​ACA​TGC​TGG​TGA​GGT-3′. STAT3: 5′-AGG​ACA​TCA​GTG​GCA​AGA​CC-3′ and 5′-CCT​TGG​GAA​TGT​CGG​GGT​AG-3′.

### Cell Proliferation Assay

Cell counting kit-8 (CCK-8) (Solarbio, CA1210) was used to assess cell proliferation. Briefly, VSMCs were seeded in 96-well plates at 5 × 10^3^/well. After being induced with PDGF-BB for 48 h, the culture medium was replaced with 100 μL fresh medium, and 10 μL CCK-8 reagent was added to wells. Then plates were kept out of light and continuously incubated at 37°C and 5% CO_2_ for 1 h in the incubator. The absorbance was detected at 450 nm by using a microplate reader (Molecular Devices, SpectraMax Plus 384) and optical density (OD) was recorded.

### Wound Healing Assay

The scratch wound healing assay *in vitro* was used to evaluate cell migration. VSMCs were seeded in 12-well plates at 6 × 10^4^/well. When cell confluency reached about 70–80%, a standard wound was made with a 200 µL micropipette tip for each well. VSMCs were then incubated for another 18 or 24 h. Images were captured under an inverted microscope (Nikon Instruments). Wound closure (%), defined as a cell coverage area in the wound divided by the total wound area, was calculated to represent migrative ability.

### Hematoxylin and Eosin Staining

Carotid arteries were excised carefully from CTR and LGL1^SMKO^ mice, fixed with 4% paraformaldehyde for 24 h and embedded in paraffin. Tissues were cut in serial 5-µm sections. After deparaffinization and rehydration, hematoxylin was used to stain the nucleus for 3 min. Differentiation was processed in 1% hydrochloric acid alcohol for 5 s. Then eosin was applied for cytoplasm and ECM staining for 2 min. The excessive stain was washed, then tissue was dehydrated in gradient alcohol and transparentized in xylene. The slides were sealed by neutral gum, and images were captured under a microscope (Nikon Instruments).

### Immunohistochemistry

After deparaffinization and rehydration, slides were immersed in antigen repair buffer (Proteintech, PR30002) and underwent a microwave thermal repair method for 20 min. Endogenous peroxide was inactivated with 0.3% H_2_O_2_ at 37°C for 10 min and non-specific antibody binding was blocked with 5% bovine serum albumin. The slides were incubated with primary antibodies at 4°C overnight. After three cycles of washing with PBS, sections were incubated with secondary antibodies at 37°C for 30 min, then diaminobenzidine as a chromogen was dropped on sections for 2–5 min. The images were viewed under a microscope (Nikon Instruments).

### Statistical Analysis

GraphPad Prism 9.0 (GraphPad Software, San Diego, CA) was used for all statistical analyses. All data are presented as mean ± SEM. The normality assumption of the data distribution was assessed by the Kolmogorov-Smirnov test. For the normal distribution, a two-tailed Student unpaired *t*-test was used to compare two groups. Differences between multiple groups with one variable were analyzed by one-way ANOVA followed by Bonferroni’s post-hoc test. *p* < 0.05 was considered statistically significant.

## Results

### LGL1 Expression was Decreased During Vascular Injury

**FIGURE 1:**
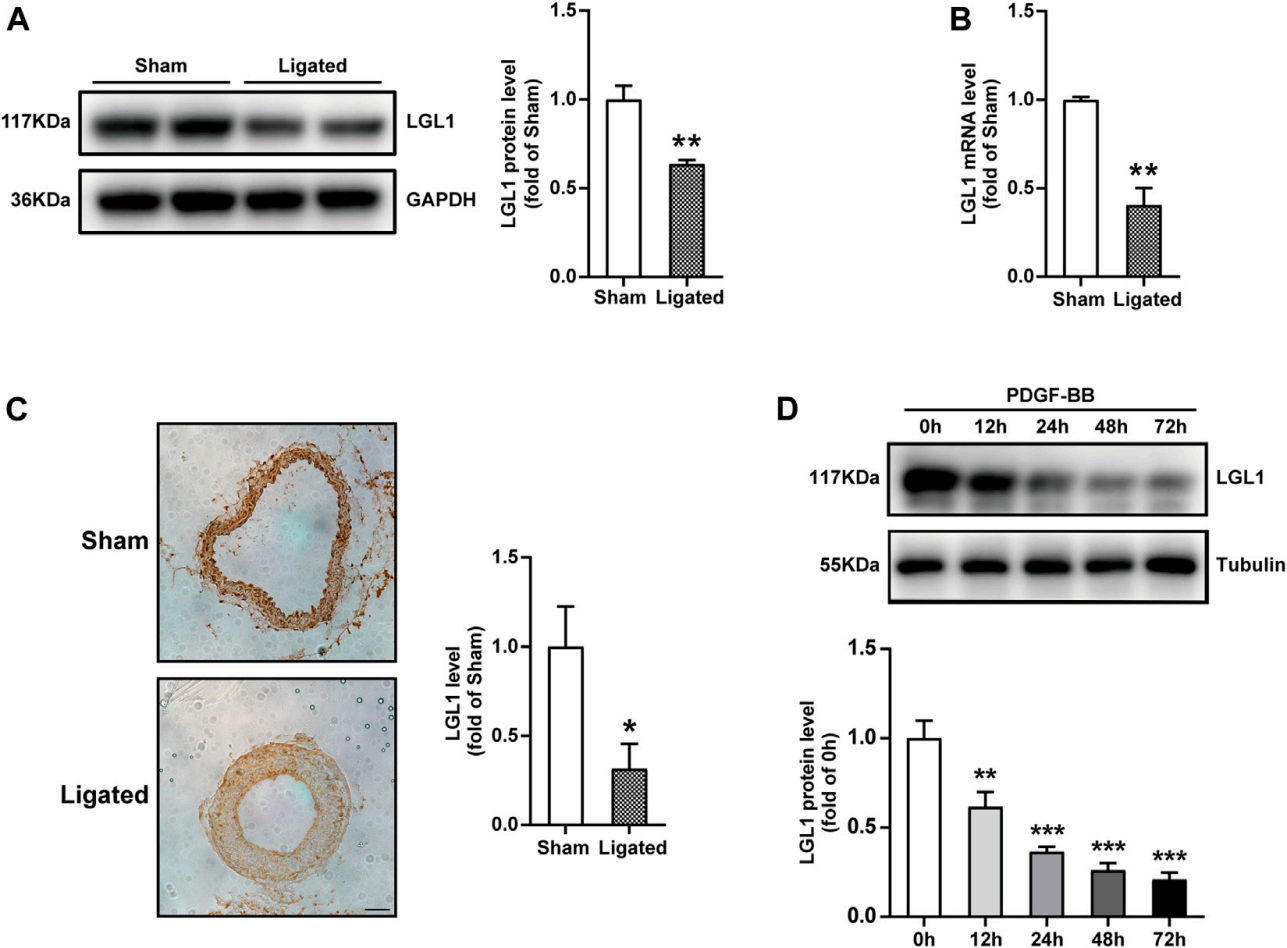
LGL1 expression was decreased during vascular injury in mice. **(A,B)** Mice underwent left CCA ligation (Ligated) to induce neointimal hyperplasia; the right CCA was a control (Sham). Protein and mRNA levels of carotid artery LGL1 were tested by western blot analysis (*n* = 4) **(A)** and qRT-PCR (*n* = 5) **(B)**. *p < 0.05 *vs.* Sham. **(C)** The expression of LGL1 from Sham and Ligated carotid arteries detected by immunohistochemistry (*n* = 4). Scale bar: 50 μm. **p < 0.01 *vs.* Sham. **(D)** VSMCs were treated with PDGF-BB (20 ng/ml) for different times, then LGL1 expression was examined by western blot analysis (*n* = 4). **p < 0.01, ***p < 0.001 *vs.* PDGF-BB 0 h.

To explore the relation between LGL1 level and vascular injury, we examined the expression of LGL1 in the mouse model of neointimal hyperplasia. The protein and mRNA levels of LGL1 were decreased in carotid arteries after ligation as compared with the sham group (Figures 1A,B). The reduced LGL1 expression was confirmed by immunohistochemistry (Figure 1C **)**. Next, VSMCs were treated with PDGF-BB (20 ng/ml), a potent stimulator of VSMCs and a key mediator in vascular injury, for different times. LGL1 expression began to decrease after 12 h of stimulation (Figure 1D). Thus, the LGL1 level was related to vascular injury, which suggests that LGL1 may be involved in the development of neointimal hyperplasia.

### LGL1 Overexpression Inhibited the Proliferation and Migration of VSMCs

**FIGURE 2:**
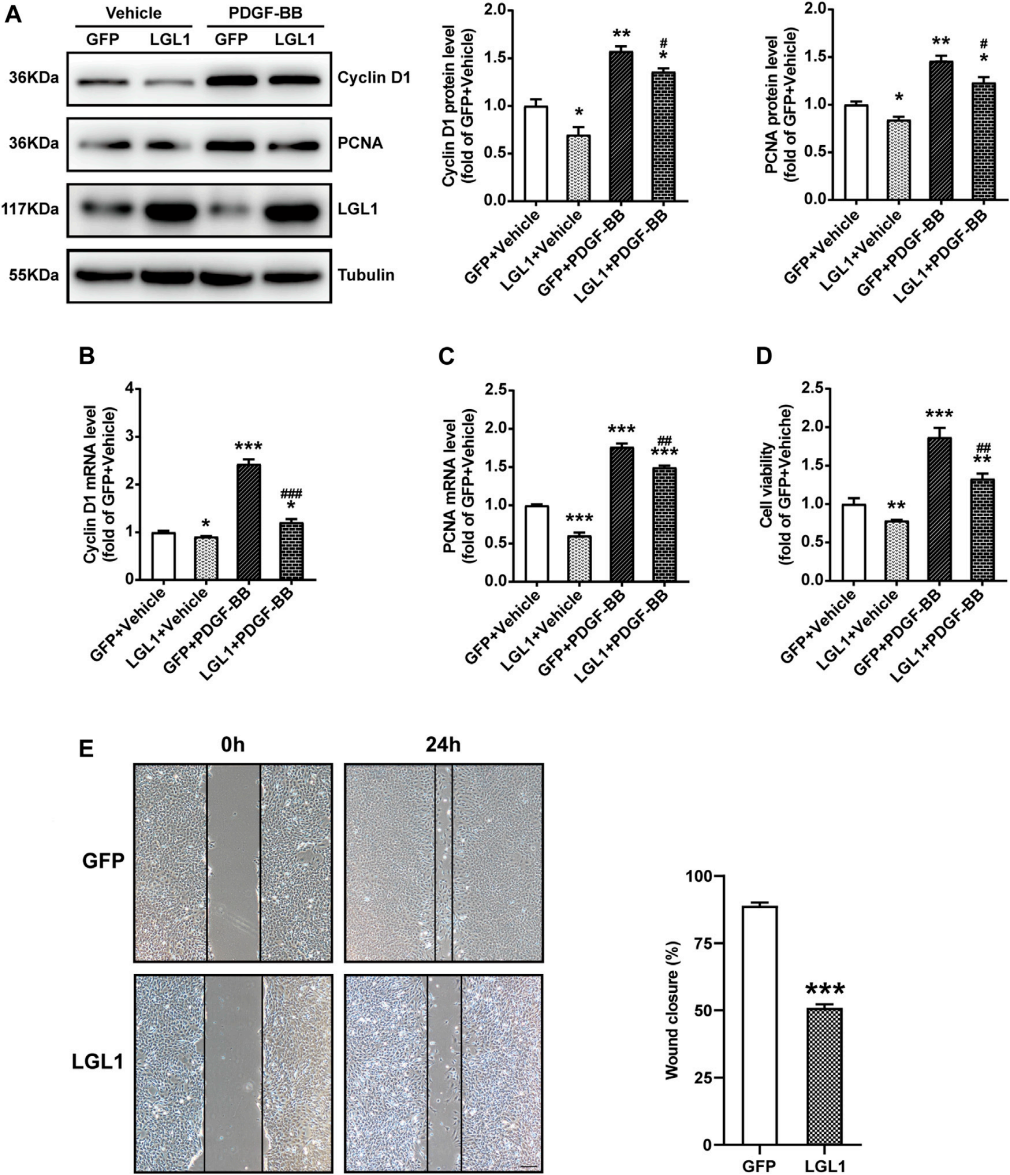
LGL1 overexpression inhibited the proliferation and migration of VSMCs. **(A)** VSMCs were infected with adenovirus-expressing GFP or LGL1, then treated with PDGF-BB (20 ng/ml) for 48 h. The protein levels of Cyclin D1 and PCNA were detected by western blot (*n* = 3). *p < 0.05, **p < 0.01 *vs.* GFP + Vehicle. ^#^ *p* < 0.05 *vs.* GFP + PDGF-BB. **(B, C)** The mRNA levels of Cyclin D1 **(B)** and PCNA **(C)** were tested by qRT-PCR (*n* = 4). *p < 0.05, ***p < 0.001 *vs.* GFP + Vehicle. ^##^ *p* < 0.01, ^###^ *p* < 0.001 *vs.* GFP + PDGF-BB. **(D)** Cell proliferation measured by CCK-8 assay (*n* = 3). **p < 0.01, ***p < 0.001 *vs.* GFP + Vehicle. ^##^ *p* < 0.01 *vs.* GFP + PDGF-BB. **(E)** Cell migration evaluated by scratch wound healing assay (*n* = 9). Wound closure (%) represented migrative ability. Scale bar: 200 μm. ***p < 0.001 *vs.* GFP.

To explore the role of LGL1 in vascular injury, VSMCs were infected with adenovirus-expressing GFP or LGL1, then treated with PDGF-BB. PDGF-BB could increase the protein expression of Cyclin D1 and PCNA, markers of cell proliferation, which was inhibited by LGL1 overexpression (Figure 2A). Similarly, PDGF-BB–upregulated Cyclin D1 and PCNA mRNA levels were also attenuated by LGL1 overexpression (Figures 2B,C). Cell viability assay revealed PDGF-BB promoted VSMC proliferation, which was suppressed by LGL1 overexpression (Figure 2D). Furthermore, on wound healing assay, LGL1 inhibited VSMC migration (Figure 2E). Hence, LGL1 overexpression inhibited the proliferation and migration of VSMCs.

### LGL1 Deletion Aggravated the Proliferation and Migration of VSMCs

**FIGURE 3:**
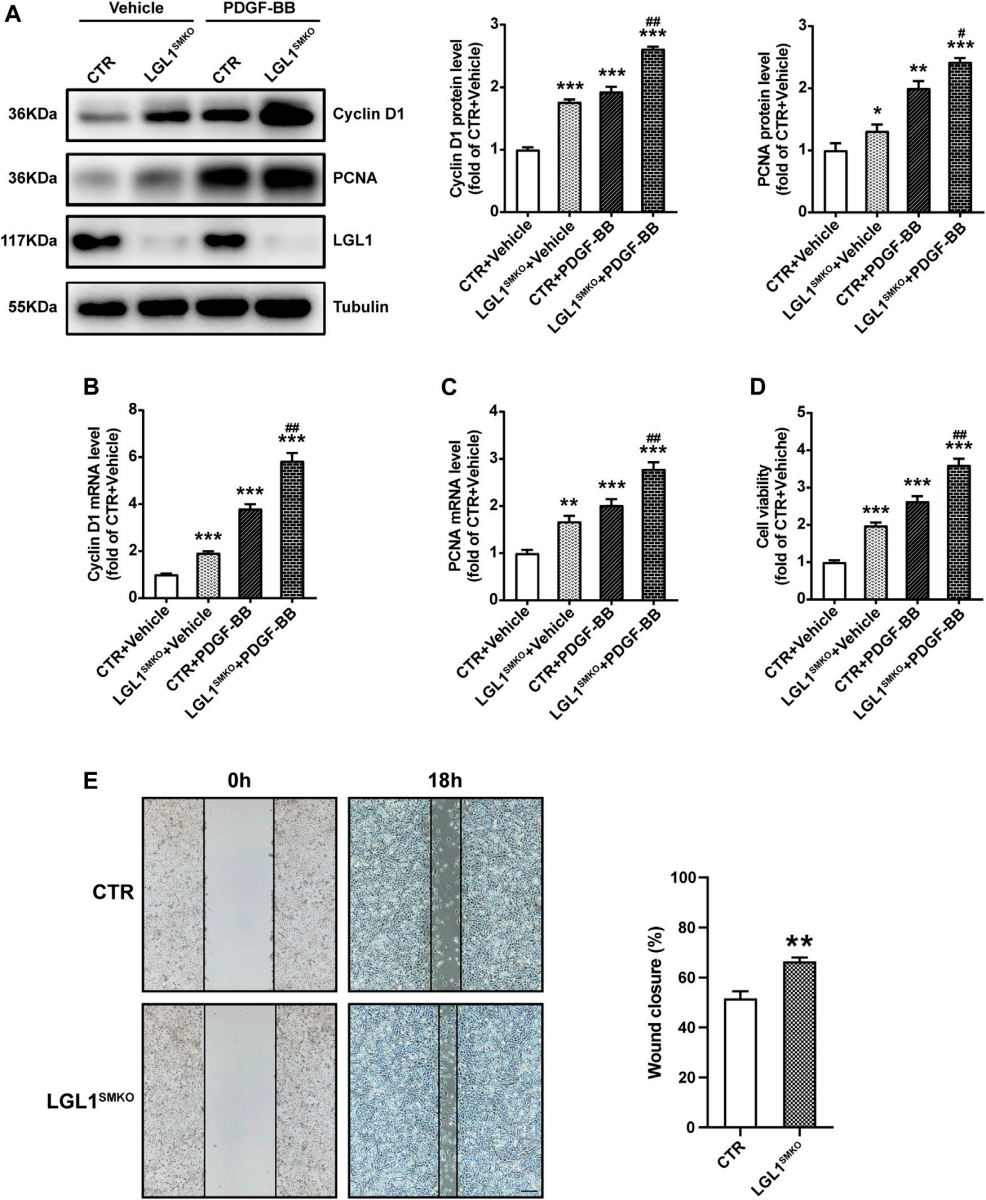
LGL1 deficiency promoted the proliferation and migration of VSMCs. **(A)** Primary VSMCs were cultured from control (CTR) and LGL1^SMKO^ mice and treated with PDGF-BB for 48 h. The protein levels of Cyclin D1 and PCNA were detected by western blot analysis (*n* = 3). *p < 0.05, **p < 0.01, ***p < 0.001 *vs.* CTR + Vehicle. ^#^ *p* < 0.05, ^##^ *p* < 0.01 *vs.* CTR + PDGF-BB. **(B,C)** mRNA levels of Cyclin D1 **(B)** and PCNA **(C)** tested by qRT-PCR (*n* = 4). **p < 0.01, ***p < 0.001 *vs.* CTR + Vehicle. ^##^ *p* < 0.01 *vs.* CTR + PDGF-BB. **(D)** Cell proliferation measured by CCK-8 assay (*n* = 5). ***p < 0.001 *vs.* CTR + Vehicle. ^##^ *p* < 0.01 *vs.* CTR + PDGF-BB. **(E)** Cell migration evaluated by scratch wound healing assay (*n* = 4). Wound closure (%) represented migrative ability. Scale bar: 200 μm. **p < 0.01 *vs.* CTR.

To comprehensively confirm the function of LGL1 in VSMCs, we cultured primary VSMCs from control and LGL1^SMKO^ mice. PDGF-BB increased the protein expression of Cyclin D1 and PCNA, which was further upregulated by LGL1 deficiency (Figure 3A). The mRNA levels of Cyclin D1 and PCNA were further augmented by LGL1 deletion under PDGF-BB stimulation (Figures 3B,C). PDGF-BB–induced VSMC proliferation was enhanced by LGL1 deficiency (Figure 3D) and LGL1 deletion promoted cell migration on wound healing assay (Figure 3E). Thus, LGL1 deficiency aggravated the proliferation and migration of VSMCs.

### LGL1 Could Bind With STAT3 and Promote its Degradation

**FIGURE 4:**
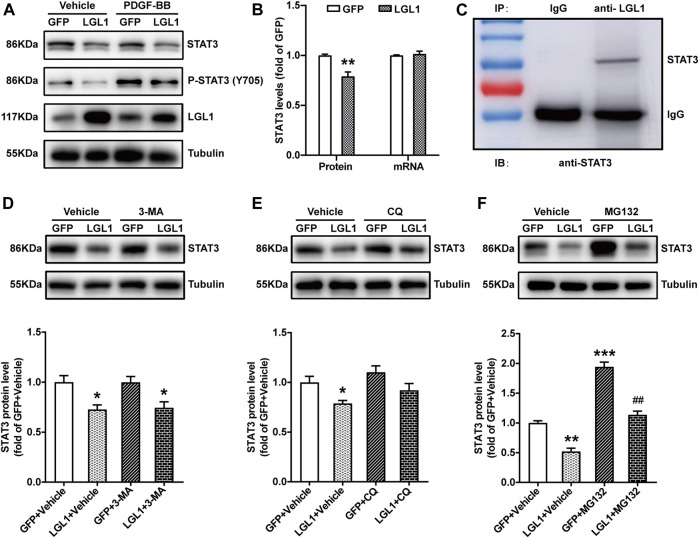
LGL1 could bind with STAT3 and promote its degradation. **(A)** VSMCs were infected with adenovirus-expressing GFP or LGL1, then treated with PDGF-BB for 15 min. The protein levels of STAT3 and P-STAT3 (Y705) were measured by western blot analysis. **(B)** Statistical analysis of STAT3 protein and mRNA levels (*n* = 4). **p < 0.01 *vs* GFP. **(C)** VSMC lysates were immunoprecipitated with IgG or anti-LGL1 antibody; STAT3 protein level was detected by western blot analysis. **(D–F)** VSMCs were infected with adenovirus-expressing GFP or LGL1, then treated with 10 mM of the autophagic inhibitor 3-MA for 24 h **(D)**, 10 μM of the lysosomal inhibitor CQ for 24 h **(E)** and 1 μM of the proteasome inhibitor MG132 for 6 h **(F)**; STAT3 protein level was detected by western blot analysis (*n* = 3). *p < 0.05, **p < 0.01, ***p < 0.001 *vs.* GFP + Vehicle. ^##^ *p* < 0.01 *vs.* LGL1 +Vehicle.

To investigate the molecular mechanism of LGL1 in regulating the proliferation and migration of VSMCs, we analyzed various pathways and related molecules. LGL1 overexpression reduced both STAT3 and P-STAT3 (Y705) protein levels (Figures 4A,B) but not STAT3 mRNA level (Figure 4B). Thus, LGL1 may affect the STAT3 protein level by regulating its degradation. Immunoprecipitation assay revealed that LGL1 could bind with STAT3 (Figure 4C). There are three pathways to promote protein degradation: autophagy, lysosomal, and proteasome pathways. VSMCs were infected with adenovirus-expressing LGL1, then treated with the autophagy inhibitor 3-MA, lysosomal inhibitor CQ or proteasome inhibitor MG132 to explore the STAT3 degradation pathway. MG132 but not 3-MA or CQ could reverse the degradation of STAT3 induced by LGL1 overexpression (Figures 4D–F). Taken together, LGL1 could bind with STAT3 and promote its degradation *via* the proteasomal pathway.

### LGL1 Inhibited the Proliferation and Migration of VSMCs *via* STAT3

**FIGURE 5:**
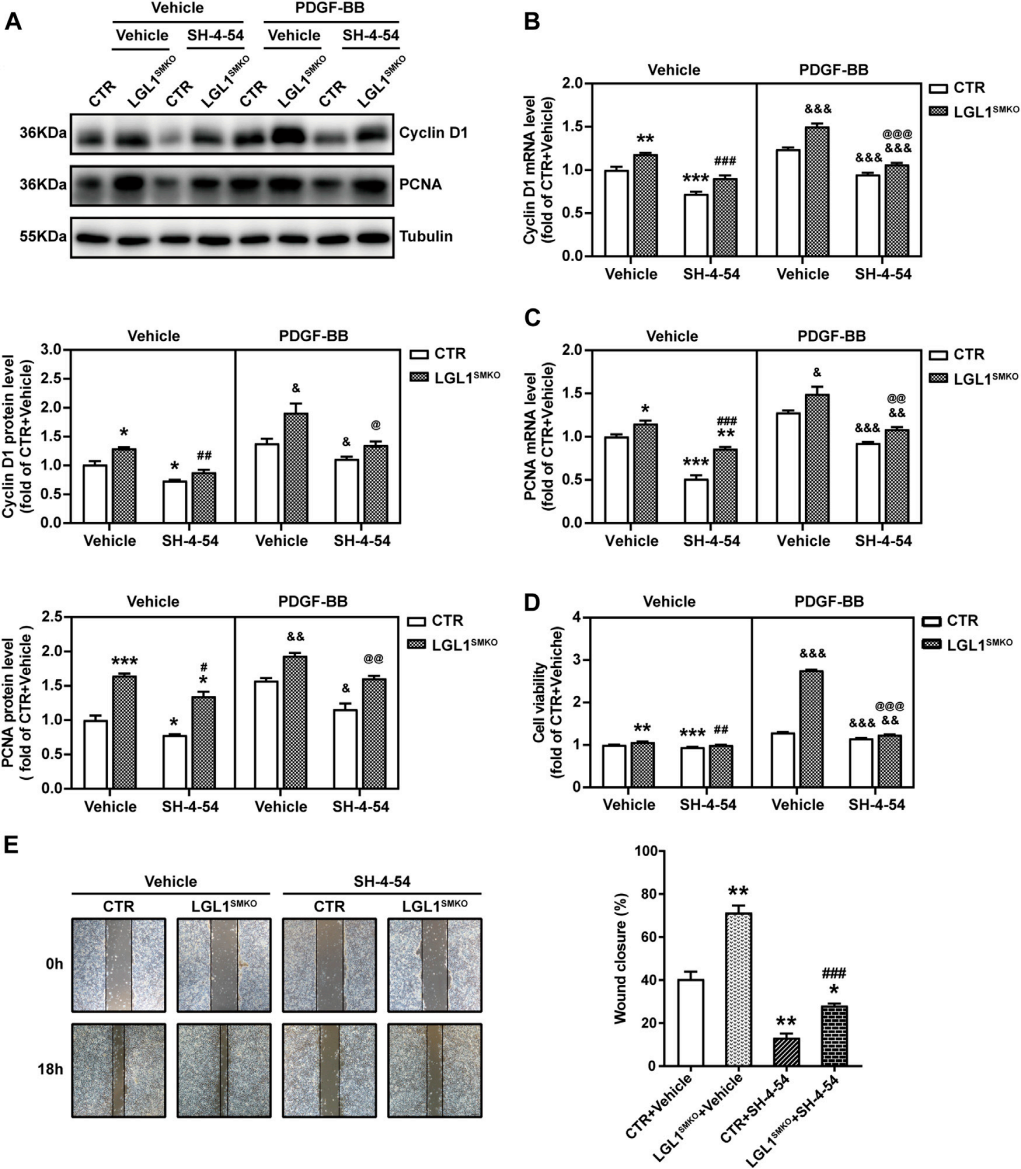
LGL1 inhibited the proliferation and migration of VSMCs *via* STAT3. **(A)** Primary VSMCs were cultured from control (CTR) and LGL1^SMKO^ mice, pretreated with or without the STAT3 inhibitor SH-4-54 at 10 μM for 24 h, and stimulated with PDGF-BB for 48 h. Protein levels of Cyclin D1 and PCNA were detected by western blot analysis (*n* = 3). **(B,C)** mRNA levels of Cyclin D1 **(B)** and PCNA **(C)** tested by qRT-PCR (*n* = 4). **(D)** Cell proliferation measured by CCK-8 assay (*n* = 8). **(E)** Cell migration evaluated by the scratch wound healing assay (*n* = 3). Scale bar: 200 μm. *p < 0.05, **p < 0.01, ***p < 0.001 *vs.* CTR + Vehicle. ^#^ *p* < 0.05, ^##^ *p* < 0.01, ^###^ *p* < 0.001 *vs.* LGL1^SMKO^ + Vehicle. ^&^ *p* < 0.05, ^&&^ *p* < 0.01, ^&&&^ *p* < 0.001 *vs.* CTR + PDGF-BB. ^@^ *p* < 0.05, ^@@^ *p* < 0.01, ^@@@^ *p* < 0.001 *vs.* LGL1^SMKO^ + PDGF-BB.

To explore whether LGL1 regulates VSMC proliferation and migration *via* STAT3, we pretreated control and LGL1-deficient VSMCs with the STAT3 inhibitor SH-4-54 followed by PDGF-BB stimulation. LGL1 deficiency increased the protein and mRNA levels of Cyclin D1 and PCNA, which were attenuated by SH-4-54 (Figures 5A–C). Consistently, LGL1 deletion promoted cell proliferation under PDGF-BB stimulation, which was significantly suppressed by SH-4-54 treatment (Figure 5D). Furthermore, LGL1 deficiency-enhanced cell migration was also attenuated by the STAT3 inhibitor (Figure 5E). Therefore, *via* STAT3, LGL1 inhibited VSMC proliferation and migration, critical processes in neointimal hyperplasia.

### Smooth Muscle-Specific Deletion of LGL1 Promoted Neointimal Hyperplasia *via* STAT3

**FIGURE 6:**
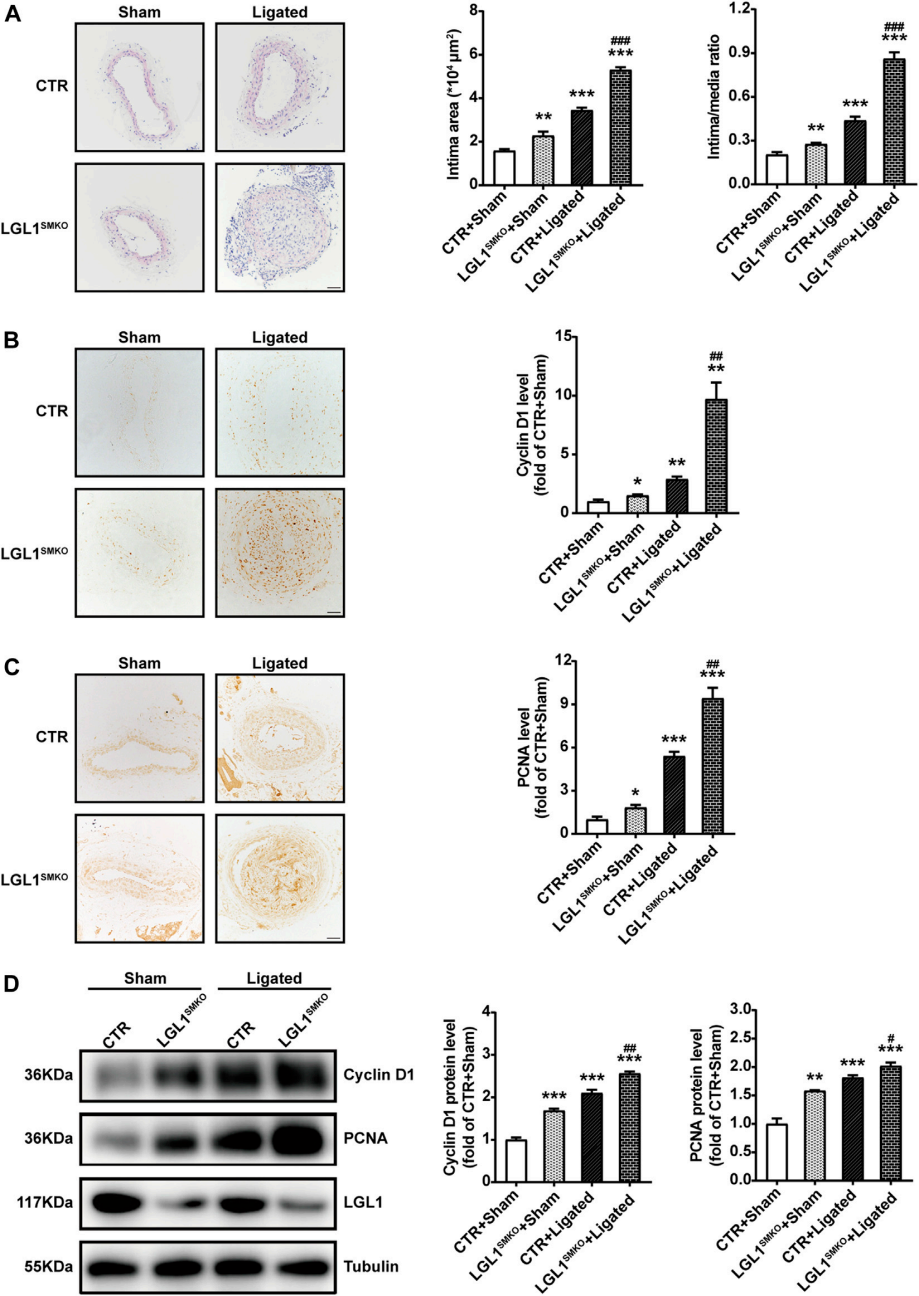
Smooth muscle-specific deletion of LGL1 promoted neointimal hyperplasia *via* STAT3. **(A)** Control (CTR) and LGL1^SMKO^ mice underwent left CCA ligation (Ligated) to induce neointimal hyperplasia; right CCA was applied as control (Sham). Carotid arteries were stained with HE. The intimal area and intima/media ratio were calculated (*n* = 6). Scale bar: 50 μm. **(B,C)** Cyclin D1 **(B)** and PCNA **(C)** levels tested by immunohistochemistry (*n* = 3). Scale bar: 50 μm. **(D)** Protein levels of Cyclin D1 and PCNA detected by western blot analysis (*n* = 4). *p < 0.05, **p < 0.01, ***p < 0.001 *vs.* CTR + Sham. ^#^ *p* < 0.05, ^##^ *p* < 0.01, ^###^ *p* < 0.001 *vs.* CTR + Ligated.

To determine the role of LGL1 in neointimal hyperplasia *in vivo*, we subjected the control and LGL1^SMKO^ mice to vascular injury by carotid ligation for 3 weeks. LGL1 deficiency significantly aggravated neointimal formation, as reflected by enlarged intima area and increased intima/media ratio (Figure 6A). Cyclin D1 and PCNA levels were increased in LGL1^SMKO^ mice, which agreed with the severe neointimal formation (Figures 6B,C). Carotid artery tissues harvested from mice post-surgery showed that ligation-induced upregulation of Cyclin D1 and PCNA was further enhanced by LGL1 deficiency (Figure 6D). Thus, smooth muscle-specific LGL1 knockout promoted neointimal formation.

### STAT3 Inhibitor Attenuated Neointimal Hyperplasia in LGL1^SMKO^ Mice

**FIGURE 7:**
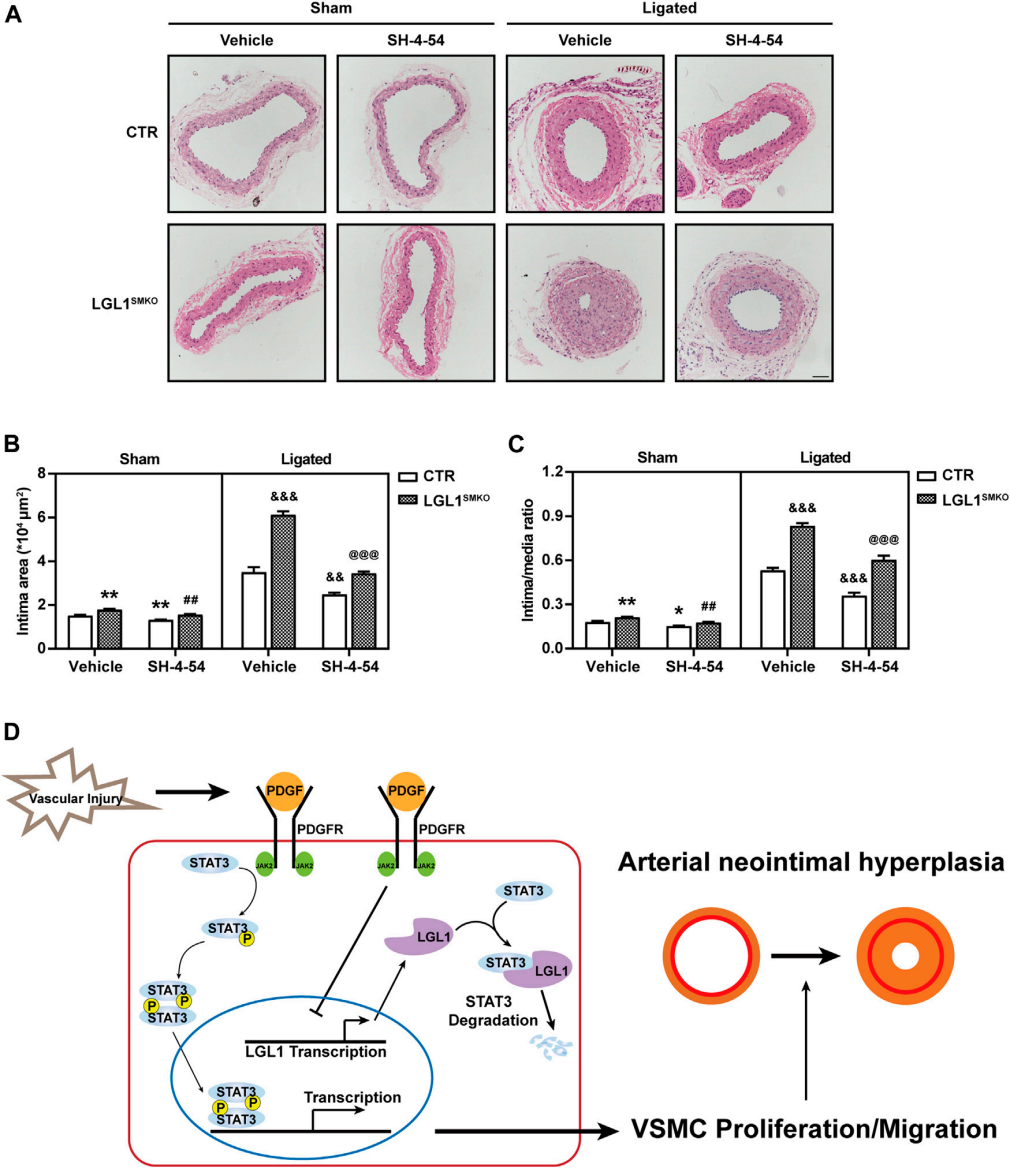
STAT3 inhibitor attenuated neointimal hyperplasia in LGL1^SMKO^ mice. **(A)** CTR and LGL1^SMKO^ mice were administrated with STAT3 inhibitor SH-4-54 (10 mg/kg) daily and then underwent left CCA ligation for 21 days. Carotid arteries were stained with HE. **(B,C)** The intimal area **(B)** and intima/media ratio **(C)** were calculated (*n* = 4). Scale bar: 50 μm. *p < 0.05, **p < 0.01 *vs.* CTR + Vehicle + Sham. ^##^ *p* < 0.01 *vs.* LGL1^SMKO^ + Vehicle + Sham. ^&&^ *p* < 0.01, ^&&&^ *p* < 0.001 *vs.* CTR + Vehicle + Ligated. ^@@@^ *p* < 0.001 *vs.* LGL1^SMKO^ + Vehicle + Ligated. **(D)** Schematic diagram of neointimal hyperplasia inhibition by LGL1 *via* STAT3 degradation.

To demonstrate whether LGL1 regulated neointimal hyperplasia *via* STAT3 *in vivo*, control and LGL1^SMKO^ mice were administrated with STAT3 inhibitor SH-4-54 and then induced to left CCA ligation for 3 weeks. Compared with control, LGL1^SMKO^ mice displayed aggravated neointimal hyperplasia, which was attenuated by SH-4-54 (Figures 7A–C). Taken together, LGL1 regulated the development of neointimal hyperplasia *via* STAT3 *in vivo.*

## Discussion

In this study, we found decreased LGL1 expression in both injured carotid arteries and PDGF-BB–induced VSMCs. To investigate the function of LGL1 in neointimal hyperplasia *in vivo*, we used smooth muscle-specific LGL1-knockout mice: LGL1 deficiency significantly aggravated neointimal formation. LGL1 overexpression inhibited PDGF-BB–stimulated proliferation and migration of VSMCs. Mechanistically, LGL1 could bind with STAT3 and promote its degradation *via* the proteasomal pathway (Figure 7D). Finally, STAT3 inhibitor treatment attenuated neointimal hyperplasia in LGL1^SMKO^ mice. Our results reveal that LGL1 inhibited neointimal hyperplasia by promoting STAT3 degradation *via* the proteasomal pathway.

LGL1, located mainly in the cytoskeleton and plasma membrane (Strand et al., 1995; Kim et al., 2005), has a crucial role in cell polarity, cell division, and differentiation (Betschinger et al., 2003; Martin-Belmonte and Perez-Moreno, 2011; Dahan et al., 2012; Zhang et al., 2015). LGL1 alters its biological activity when phosphorylated by atypical protein kinase C (aPKC) (Graybill and Prehoda, 2014). Conjugated with Par/Cdc42/aPKC, LGL1 joins in the complex to regulate cell polarity and membrane development (Plant et al., 2003; Tocan et al., 2021). In addition, LGL1 in mammals acts as a tumor suppressor in many types of cancer progression (Tsuruga et al., 2007; Lu et al., 2009; Song et al., 2013; Liu et al., 2015). Moreover, LGL1 deficiency in the nervous system caused disrupted asymmetric cell division and lack of differentiation and hyperproliferation to apoptosis in progenitor cells, and mice developed tumors or severe brain dysplasia (Klezovitch et al., 2004; Daynac et al., 2018). Interestingly, the chimeric mice with a hematopoietic system deficient for LGL1 showed a stronger antiviral and antitumor effector CD8^+^ T-cell response, which resulted in enhanced control of MC38-OVA tumors (Ramsbottom et al., 2016). Our recent study explored the role of LGL1 in vascular disease. LGL1 inhibited osteogenic differentiation by promoting degradation of high mobility group box protein 1 in vascular calcification (Zhang et al., 2020). In this study, we found that the protein and mRNA levels of LGL1 were decreased in carotid arteries after ligation, which indicates that vascular injury inhibited LGL1 expression at the transcriptional level. Moreover, LGL1 could inhibit neointimal hyperplasia after injury, which amplified the biological function of LGL1.

STAT3 plays an important role in many pathological processes. When cells are stimulated by interleukin families, growth factors, angiotensin, erythropoietin, and colony-stimulating factors, tyrosine kinase-associated receptors in the cell membrane transduce the signal to tyrosine kinase (JAK), which phosphorylates STAT3 at Tyr705. Phosphorylated STAT3 dimers translocate into the nucleus to regulate the expression of target genes (Aggarwal et al., 2009; Brooks et al., 2014). STAT3 phosphorylation at Ser727 could increase the binding stability of DNA with STAT3 and augment its transcriptional activity (Yang et al., 2002). Acetylation at Lys685 was critical for STAT3 dimerization and transcriptional regulation (Yuan et al., 2005). SUMOylation at Lys451 caused the hyperphosphorylation of STAT3 and magnified its transcription activation (Zhou et al., 2016). Also, PIAS3, known as protein inhibitor of activated STAT3, could block the DNA-binding activity of STAT3 and inhibit STAT3-mediated gene expression (Chung et al., 1997). Moreover, the expression of STAT3 could be modulated by transcription factors such as PPARγ, Src and SMAD3, or microRNAs (miRNAs) such as miR-125a-5p and miR-519a (Xiao et al., 2017; Li et al., 2018; Xu et al., 2018; Liu et al., 2021; Zhang et al., 2021). In addition, some small molecules such as SD-36 could degrade STAT3 to disrupt its biological function (Bai et al., 2019; Zhou et al., 2019). In this study, we found that LGL1 could bind with STAT3 and promote its degradation *via* the proteasomal pathway. In our previous study, we demonstrated that LGL1 could inhibit vascular calcification by preventing osteogenic differentiation through degrading HMGB1 in the lysosomal pathway (Zhang et al., 2020). These results indicated that LGL1 might mediate the degradation of proteins in different ways. The detailed mechanisms will be explored in future study. Moreover, the STAT3 inhibitor SH-4-54 attenuated the aggravated neointimal hyperplasia in LGL1^SMKO^ mice, which suggests that STAT3 may be a target for preventing and treating vascular diseases.

Neointimal hyperplasia is a complicated process referring to various cells and cellular cytokines. In response to injury, inflammatory cells along with platelets and fibrin recruit immediately around the impaired vascular surface and secrete cytokines such as PDGF-BB, which propel quiescent VSMCs in the tunica media to proliferate and migrate into the intima (Scott, 2006; Kim and Dean, 2011). The dysfunctional endothelium with released active mediators and degraded ECM induce VSMCs to transform from a “contractile” to a “synthetic” phenotype, which is more mobile and productive (Ip et al., 1990; Docherty et al., 1992; Scott, 2006; Park et al., 2020). During the process of neointimal hyperplasia, VSMCs are predominant. Inhibition of the proliferation and migration of VSMCs could be a promising strategy to treat neointima-related diseases such as atherosclerosis. In this study, we demonstrated that LGL1 could inhibit the proliferation and migration of VSMCs, which attenuated neointimal formation and increased our understanding of the mechanism of neointimal hyperplasia. Besides, in our previous study, we demonstrated that LGL1 could inhibit vascular calcification by preventing osteogenic differentiation through degrading HMGB1. These findings disclosed the vital role of LGL1 in vascular remodeling, which suggests that LGL1 may be the potential therapeutic target in the vascular remodeling-related diseases such as atherosclerosis.

In conclusion, we revealed that LGL1 could inhibit neointimal hyperplasia after vascular injury: it suppressed VSMC proliferation and migration by promoting STAT3 degradation *via* the proteasomal pathway. Our findings may shed light on the mechanism of neointimal formation and provide a novel strategy to treat vascular remodeling diseases.

## Data Availability

The original contributions presented in the study are included in the article/Supplementary Material, further inquiries can be directed to the corresponding authors.
